# Characterization of the Jet-Flow Overpressure Model of Traumatic Brain Injury in Mice

**DOI:** 10.1089/neur.2020.0020

**Published:** 2021-01-05

**Authors:** Min-Kyoo Shin, Edwin Vázquez-Rosa, Coral J. Cintrón-Pérez, William A. Riegel, Matthew M. Harper, David Ritzel, Andrew A. Pieper

**Affiliations:** ^1^ Harrington Discovery Institute, University Hospitals Cleveland Medical Center, Cleveland, Ohio, USA.; ^2^ Department of Psychiatry and Department of Neuroscience, Case Western Reserve University, Cleveland, Ohio, USA.; ^3^ Geriatric Research Education and Clinical Centers, Louis Stokes Cleveland VAMC, Cleveland, Ohio, USA.; ^4^ Stumptown Research and Development, LLC, Black Mountain, North Carolina, USA.; ^5^ Center for the Prevention and Treatment of Visual Loss, Veterans Affairs Medical Center, Iowa City, Iowa, USA.; ^6^ Departments of Ophthalmology and Visual Sciences, University of Iowa, Iowa City, Iowa, USA.; ^7^ Dyn-FX Consulting, Ltd., Amherstburg, Ontario, Canada.

**Keywords:** blast, jet flow, multi-modal traumatic brain injury, overpressure chamber

## Abstract

The jet-flow overpressure chamber (OPC) has been previously reported as a model of blast-mediated traumatic brain injury (bTBI). However, rigorous characterization of the features of this injury apparatus shows that it fails to recapitulate exposure to an isolated blast wave. Through combined experimental and computational modeling analysis of gas-dynamic flow conditions, we show here that the jet-flow OPC produces a collimated high-speed jet flow with extreme dynamic pressure that delivers a severe compressive impulse. Variable rupture dynamics of the diaphragm through which the jet flow originates also generate a weak and infrequent shock front. In addition, there is a component of acceleration-deceleration injury to the head as it is agitated in the headrest. Although not a faithful model of free-field blast exposure, the jet-flow OPC produces a complex multi-modal model of TBI that can be useful in laboratory investigation of putative TBI therapies and fundamental neurophysiological processes after brain injury.

## Introduction

Traumatic brain injury (TBI), including mild TBI, commonly occurs in crashes, falls, explosions, and assaults, and often results in chronic neuropsychiatric disorders.^1–4^ Within the United States alone, the annual incidence of TBI is estimated at ∼3.5 million cases, and worldwide it is estimated to be as high as 50 million.^5,6^ Moreover, at least 3.2 to 5.3 million Americans currently live with one or more TBI-related disabilities, resulting in an estimated $80 billion in annual direct costs and an additional $65 billion in lost productivity.^7,8^

Patients with TBI experience an increased prevalence of many progressive and debilitating conditions, including visual deficits, chronic pain and fatigue, headache, post-traumatic stress disorder, major depressive disorder (MDD), and neurodegenerative disease.^9^ Indeed, one of the most prominent complications of TBI is increased risk of dementia (relative risk [RR] = 1.63), especially Alzheimer's disease (AD; RR = 1.51),^10^ and TBI has been epidemiologically identified as one of the strongest non-genetic, non-age-related risk factors of AD.^11–15^ In addition, new onset MDD, frequently the earliest presenting symptom in AD,^16^ is also increased after TBI,^17^ along with physical aggression and suicidal thoughts and behavior.^18^ There are currently no protective pharmacological treatments that stop neurodegeneration after TBI.

TBI is highly prevalent in the military, with more than 250,000 cases reported between 2000 and 2012,^19–23^ a great many of which are due to blast-mediated TBI (bTBI). Apart from the blast effect of explosive munitions and improvised explosive devices (IEDs) during active combat, bTBI is also suspected of being inflicted by repeated exposure to operational weapon noise during training, such as from muzzle blast or explosive breacher operations.^24,25^ Thus, bTBI has emerged as the signature injury of our modern military, with a current incidence of ∼20% in combat veterans.^26–29^

In contrast to its dismissal as “shell shock” in earlier wars,^30^ the unique aspects and increasing prevalence of bTBI from the recent Operation Enduring Freedom/Operation Iraqi Freedom/Operation New Dawn conflicts are now being acknowledged widely. In efforts to study and develop treatments for bTBI, scientists have attempted to develop relevant laboratory models. However, this has not traditionally proceeded via a multi-disciplinary approach that draws on collective expertise from medicine, neuropsychiatry, and blast physics. As a consequence, most bTBI models in laboratory use today have failed to incorporate basic blast-specific biomechanics. Indeed, the majority of reported laboratory bTBI models fail to faithfully recapitulate conditions of free-field blast exposure, including the jet-flow overpressure chamber (OPC) that we and others have used.^31–41^ We describe here a rigorous and comprehensive physical characterization of the jet-flow OPC, which reveals that this apparatus instead produces a model of multi-modal TBI (mmTBI) that is useful for the field yet different in nature from what was previously assumed. We also summarize the important features of blast physics that must be incorporated into an accurate laboratory model of bTBI.

## Methods

### Jet-flow OPC apparatus

**FIG. 1.**
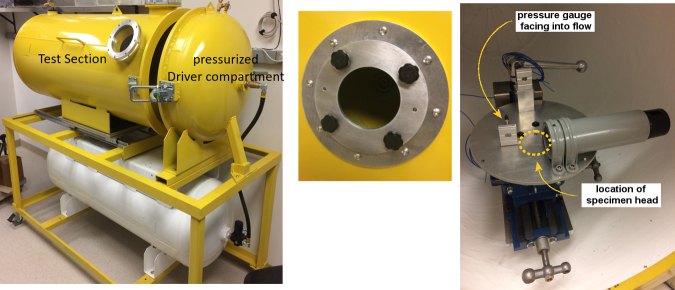
This is a photograph of the principle components of the jet-flow overpressure chamber. On the left is shown the close-ended Test Section into which the animal is placed, and the Pressurize Drive Compartment into which compressed air is delivered in order to burst the membrane. In the middle is shown the diaphragm where the Mylar membrane is placed, between the two chambers. On the right is shown the holder into which the animal is placed.

The jet-flow OPC apparatus (Fig. 1) consists of a steel vessel 0.6 m in diameter and 1.8 m long with a partition between a high-pressure Driver Section and a Test Section. In normal operation, a rodent specimen is mounted within a canister such that the head is exposed and supported by a backrest. The specimen canister is positioned on a platform in the Test Section ∼12.7 cm from an aperture 14 cm in diameter in the partition wall separating the Test and Driver Sections. This aperture is fitted with a frangible plastic diaphragm (Mylar A material; DuPont Reijun Film U.S. Ltd. Partnership, Hopewell, VA, USA) designed to rupture at prescribed compressed-air generated Driver pressures. On the platform, a pressure gauge mounted on a small plate facing the incoming flow over the platform measures exposure conditions. This sensor measures the stagnation or total pressure conditions of the incoming flow, which is partially disrupted by the test platform.

Diaphragm membrane fragments can damage sensors as well as cause signal anomalies for sensors in the “face-on” orientation, such as typically used for stagnation pressure measurements. Such fragments may also lacerate the specimen if it is sufficiently close. However, Mylar membranes such as those used in this study do not shatter upon bursting. Instead, Mylar membranes tear along radial lines from the center, such that the membrane petals open with the pieces remaining attached at the clamping perimeter. Thus, the torn membrane remains in one piece and does not travel into the Test Section.

To record, review, and analyze via digital signal processing, an instrumentation suite of pressure sensors and a data acquisition system were installed within the jet-flow OPC (AstroNova TMX, at a sampling rate of 800 kHz on all channels). Preliminary tests were conducted using this instrumentation suite with the standard experiment configuration, including a test table with canister for mounting the specimen. The new pressure sensor and recording system was also used for the measurement taken at the mounting plate, as shown in Figure 1. A high-performance pressure sensor was used to monitor conditions in the Driver chamber (Endevco 8530C-100, Meggitt Sensing Systems, Irving, CA, USA). This preliminary test series confirmed that the new instrumentation system reproduced prior results from the same chamber, such that review of the records from the Test Section in conjunction with the time-resolved conditions in the Driver Section would be relevant. Specifically, two test series were conducted at the same nominal diaphragm-burst conditions of 138 kPa (20 psi), involving a total of 23 tests to confirm reproducibility of results.

**FIG. 2.**
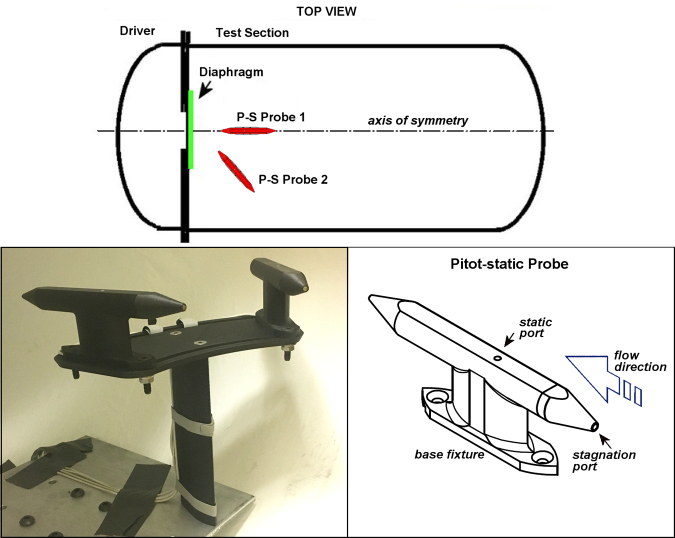
Configuration of the Pitot-static probes installed for analyses of free-field flow conditions generated by the jet-flow overpressure chamber.

In the first test series, the entire specimen platform was removed from the Test Section to allow installation of specially made Pitot-static probes (PPP2001, Stumptown Research and Development, LLC) to measure the required free-field (unobstructed flow) conditions (Fig. 2). The Pitot-static probe is aerodynamically designed with a pressure port at the tip facing into the flow to measure stagnation or total pressure, and a port along the side to measure the static or “side-on” pressure. These combined measurements allow determination of key flow conditions, including dynamic pressure. Two Pitot-static probes were installed as shown in Figure 2, one on-axis with the expected jet flow at the nominal location of the specimen head, and the other at the same distance from the venting orifice but positioned 45 degrees to the jet-flow axis. Thus, the stagnation (front tip) port of Probe 1 was positioned to measure conditions close to those of the gauge mounted in a block during normal experiments with a specimen, without the flow disruption that would normally be caused by the table and specimen canister.

The probes were constructed of three-dimensional (3D)-printed nylon and chopped carbon-fiber mix, with continuously layered strand carbon-fiber reinforcement. The sensors were Endevco 8530C-100s, which are piezoresistive 100-psi absolute pressure transducers. The probes and probe mounts were specially made to be mounted within the Test Section without impeding the flow. The mounting system also allowed for measurements to be taken at multiple angles relative to the jet flow, which is difficult to accomplish with traditional pitot probes and mounting methods. Although the probes are not made of aluminum as are most pitot probes, the carbon-fiber inlays provide a stiffness comparable to AL-6061. In comparison testing of the carbon-fiber reinforced probes versus AL-6061 probes performed by Stumptown Research and Development, LLC, there was no difference in measurements during testing.

In the second test series, high-speed video (HSV) at framing rates between 8 and 9 kfps was used to observe and analyze the flow conditions in the Test Section by various flow visualization techniques. The HSV camera (Chronis 1.4, Kron Technologies) was positioned externally to the viewport as shown in Figure 1, with a high-intensity LED light bar secured internally to the wall of the Test chamber. Flow visualization methods included seeding locations with talc-dust to monitor flow patterns, as well as tracking “free-flight” spheroids to assess the accelerating forces on objects. HSV imaging was also used to monitor the details of the diaphragm rupture.

*S*pheroids were table-tennis balls adapted for this demonstration by (1) injection with gelling compound to provide ballast mass and (2) application of surface tape to add roughness. The size, mass, and surface texture were chosen to approximate the flow Reynolds number relevant to an object the scale of a small rodent, an important parameter for scaling in fluid dynamics. Note that all flow phenomena of this type are optically invisible. This demonstration was simply intended to allow the reader to visualize the effect of the flow forces involved, which would otherwise not be evident.

## Results

**FIG. 3.**
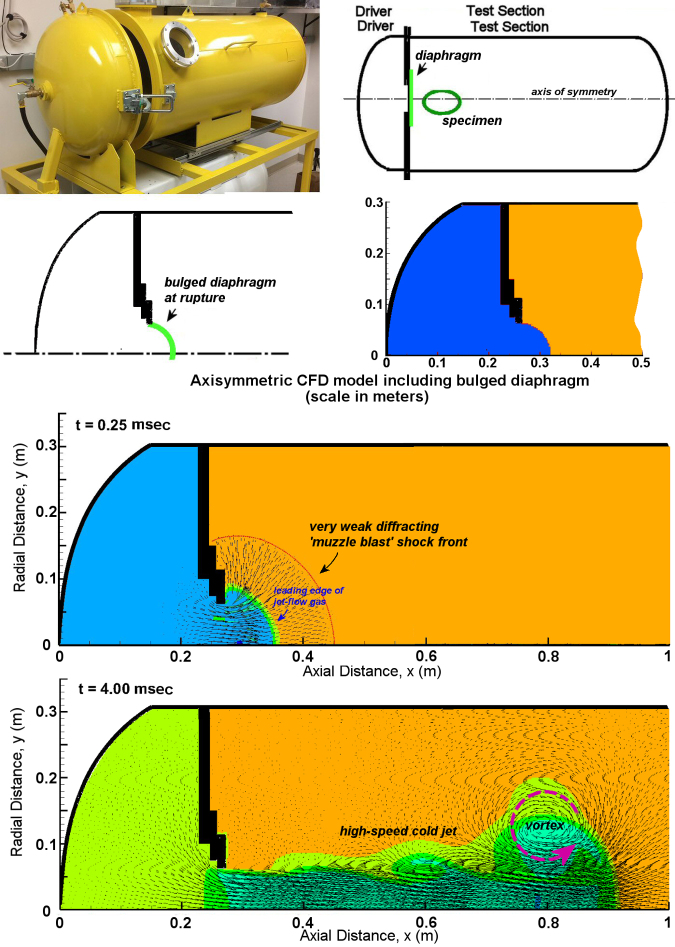
Illustration of the computational fluid dynamics model of the jet-flow overpressure chamber. Animation of the jet-flow development is presented in Supplemental Figure S1.

We began by using numerical modeling to predict flow conditions via a computational fluid dynamics (CFD) Chinook code that was specialized for blast problems, to enable visualization and resolution of gas dynamics.^43^ The shape of the apparatus allowed the modeling to be simplified to two-dimensional (2D) axisymmetric geometry as shown in Figure 3. The bursting of the bulged diaphragm was revealed to initially produce a weak, spherically expanding precursor shock, termed a wavelet (Fig. 3). The energy of this initial burst was due to the pressurized volume within the hemispherical bulge of the diaphragm and equivalent to about 40 mg of explosive.

At the specimen location, the precursor shock wavelet was found to be ∼20 kPa (3 psi) in amplitude, with an ∼0.2 msec duration. However, once this early event was complete, the pressurized gas within the Driver Section was released as collimated jet flow, which penetrated into the Test Section as a high-speed column of gas. This jet flow decayed rapidly in strength over 18 msec as the Driver Section depressurized. While the head of the jetting column of gas accelerated to ∼150 m/sec, the flow velocities within the jet itself briefly exceeded 450 m/sec, which is supersonic. This extreme peak velocity was due largely to the effect of a ring vortex, as shown in Figure 3. A ring vortex of this type is typically generated by abruptly initiated venting flow. In this case, the vortex followed closely behind the head of the jetting gas stream emerging from the Driver Section, and was directly related to peak levels in flow velocity and dynamic pressure as it passed. Simultaneously, this also caused partial vacuum conditions in static pressure (Fig. 3).

In addition, the jetting gas of expanded air from the Driver Section was initially pressurized to ∼138 kPa or 20 psi, and was found to be extremely cold and dense due to its expansion with a minimal temperature around −60°C. Here, we selected piezoresistive sensors, because they are far less sensitive than piezoelectric sensors to thermal drift and acceleration. We carefully monitored records for adverse effects, such as thermal drift, by attention to the late-time response. This parameter consistently correctly converged on the same true quasi-static pressure, slightly above laboratory ambient level as expected. We also note that the thermal insult within the jet stream is extremely brief (<20 msec), with only the peak deficit being −60°C. Therefore, there is negligible time for heat transfer for an object with the thermal mass of a rodent. Although the temperature within the chamber was not measured, the excellent correlation with CDF permits application of those temperature results with confidence. The main physiological effect of the cold jet temperature is to increase density of the jet flow, which has an immediate specimen effect on the stagnation pressure loading.

**FIG. 4.**
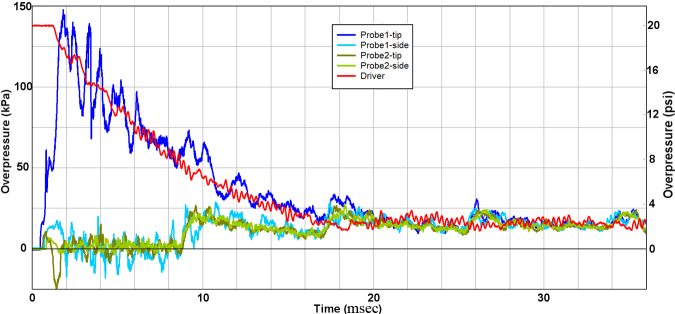
Representative experimental records from current work.

Representative measurements from the first test series are shown in Figure 4, confirming the flow prediction from the CFD study shown in Figure 3. Notably, after a short start-up period before the jet flow fully developed, the stagnation measurement from the tip of Probe 1 directly followed the decay of pressure within the reservoir for about 1.5–18 msec. The tip sensor on the probe measured the total flow pressure, meaning the combined effect of static and dynamic pressure. However, the static pressure was negligible over this period, including frequent excursions into partial vacuum conditions. Thus, the damaging energy within this jet was almost entirely due to the kinetic energy of the flow, also known as the “blast wind.” The stagnation pressure from Probe 1 did not decay to ambient level, but instead eventually equilibrated to about 15 kPa.

Because of the finite volume of the Test Section, the release of pressurized gas from the Driver Section into the Test Section ultimately caused the combined volume of the Driver and Test Sections to equilibrate to pressure slightly above ambient level. It is important to note that all pressure sensors converged to the final quasi-static level of about 15 kPa in stages directly related to the timing of a weak longitudinal compression wave, which reverberated along the length of the Test Section with a period of about 8.5 msec/cycle. The compression wave itself was thus due to the global effect of the abrupt intrusion of jet-flow gas from the Driver Section into the Test Section. Measurements from Probe 2 confirmed that only the minor precursor wavelet of ∼10 kPa and ∼0.0.3 msec duration was detected off-axis from the jet flow.

Although our CFD modeling showed the weak precursor shockwave to be hemi-spherical, the diaphragm actually ruptured in a non-ideal manner. Specifically, the plastic film usually split first at the center and then petaled outward over ∼0.3 msec. Because the probes were positioned very close to the bulging diaphragm, this non-ideal diaphragm rupture affected early flow development, and thus explains the differences and irregularities of the flow conditions between the two probes within the first 2 msec. Over the longer time span of tens of milliseconds, both the stagnation and static measurements from Probe 2 followed the quasi-steady pressurization of the Test Section volume, as expected. Importantly, the non-ideal rupture dynamics of the membrane are such that the animal would be subjected to a true shock front in perhaps only 10% of tests. Such a shock wavelet would be very weak and have a duration of a small fraction of a millisecond.

**FIG. 5.**
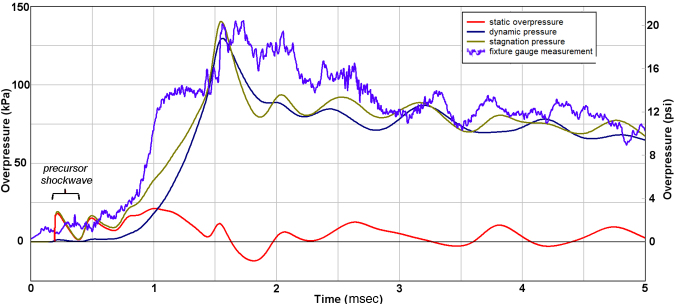
Comparison of experimental records with computational fluid dynamic modeling for the early flow development and peak levels.

Next, we compared pressure measurements at the specimen location with the CFD prediction (Fig. 5). This further confirmed that prior measurements from the gauge station on the platform near the specimen were recording the time-decay of stagnation conditions of the jet flow, rather than the static overpressure of a passing shockwave. In fact, the static overpressure was found to fluctuate at a low level below 20 kPa throughout the period of jet-flow decay. There was also close agreement between measurement and CFD prediction, including weak fluctuations in the pressure decay having a frequency of ∼1.3 kHz (Fig. 5). These weak oscillations were due to radial reverberation of the rare faction wave in the Driver Section, which caused mild pulsing of the gas jet efflux. Importantly, any non-ideal rupture of the diaphragm would make these perturbations more severe.

The jet flow itself was also noted to be supersonic and locally 2D as the vortex passed, yielding peak levels in stagnation pressure between 1.5 and 2 msec (Fig. 5). This computed peak flow was based on the Bernoulli-flow assumption that stagnation pressure is the sum of static and dynamic pressure. Correction for the brief period of supersonic flow was considered unnecessary for the current objectives to confirm the basic character of the flow in comparison with that of explosive blast.

**FIG. 6.**
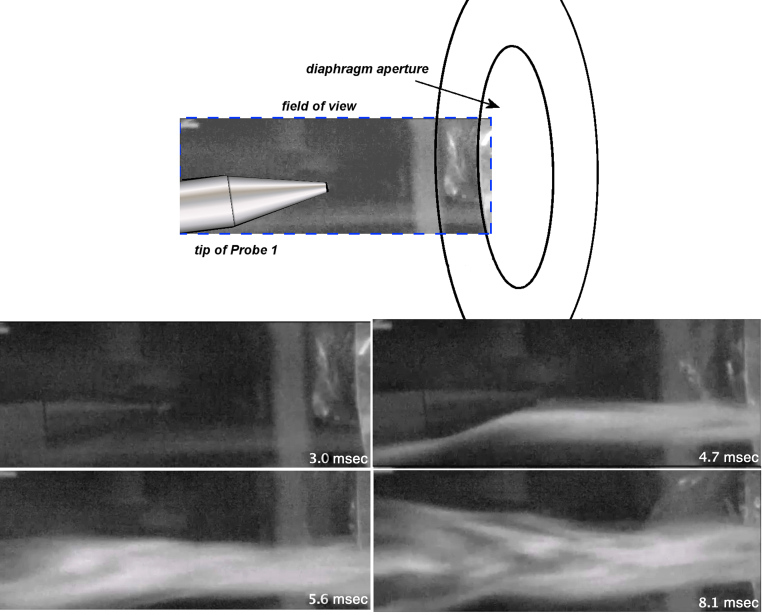
Frame sequence from high-speed imaging of jet-flow developed at the nominal specimen location. For purposes of referencing time relative to the bursting of the diaphragm, 0.11 ms would be added to each time shown. High-speed video is presented in Supplemental Figure S2.

**FIG. 7.**
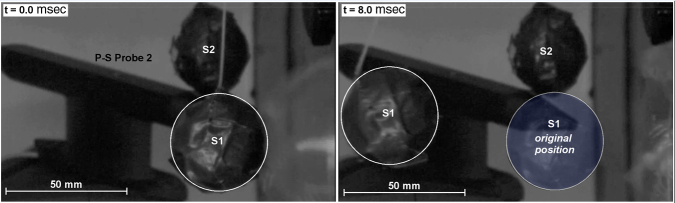
Frame sequence from high-speed imaging of the free-flight motion of a ballasted sphere subjected to the jet-flow impingement. The test sphere attained a velocity of about 15 m/s within 5 ms or an acceleration about 270*g*.

Sample results from the second test series concentrating on flow analyses by means of HSV imaging are presented in Figures 6 and 7. In Figure 6, results are shown from particle-tracking dust that was seeded in the diaphragm aperture separating the Driver and Test Sections. Upon diaphragm rupture, a well-defined collimated jet of gas was observed streaming from the Driver Section, which gradually decayed over ∼20 msec. Importantly, the jetting behavior and timescale were consistent with the CFD flow prediction.

Next, to illustrate loading effects from the jet-flow OPC, a 40-mm ball ballasted to a density of 1.1 gm/cm^3^ was set at the specimen location immediately above the tip of Probe 1, as shown in Figure 7. The suspended spheroid S1 was free to move under loading, such that tracking its motion allowed estimation of the applied forces and their direction. Upon diaphragm rupture, spheroid S1 moved only ∼1 mm over the first millisecond, but then rapidly accelerated to a velocity of ∼15m/sec over the next 4 msec as the jet flow fully developed. The impulse imparted for this period was estimated to be 0.57 N/sec, yielding an acceleration of about 270*g*. Although in normal testing the animal's head is restrained from excessive motion by a backrest, it is clear that the impulsive force demonstrated for spheroid S1 will be similar and cause the head to be agitated somewhat and forcefully compressed against the backrest. Next, a second free-flight spheroid S2 was placed over the tip of Probe 2, located 45 degrees to the axis of the jet-flow stream (Fig. 7). Notably, spheroid S2 did not move during the entire duration of the test, as expected from the pressure records at Probe 2, which was outside the jet-flow stream and was subjected to only the weak precursor shock wavelet.

## Discussion

Our results show that the jet-flow OPC, which has been widely used in experimental studies designed to model bTBI,^31–41^ actually generates a powerful, collimated jet flow of very high dynamic pressure at the specimen location. Although the intensity of this jet flow decays over 18 msec as the Driver Section depressurizes, this exposure condition does not represent that of explosive blast or a traveling shockwave.^44,45^ Whereas exposure to high-speed jet flow can elicit neurotrauma, the imparted forces are different from those of an explosive blast wave, particularly with regard to the conditions of static and dynamic pressure. Indeed, the fundamental physics of a jet-flow apparatus is that the high static pressure within the Driver Section is converted to dynamic pressure (kinetic energy) at the venting aperture, which is created by diaphragm rupture. This process generates a powerful collimated jet-stream of gas with extreme dynamic pressure (high kinetic energy), yet negligible or partial vacuum conditions for static pressure. Thus, the jet-flow OPC is not a model of free-field blast exposure.

This model may be termed multi-modal TBI (mmTBI), with the caveat that this is purely a descriptive term for the experimental condition, and not a term that is currently used in clinical diagnosis of human TBI. We note, however, that this model is clearly capable of inducing neurotrauma^31–41^ and may be considered useful for studying brain cell processes after injury and in evaluating the efficacy of putative therapeutic agents. For example, through this mmTBI model we have recently reported the unexpected ability to reverse chronic neurodegeneration in the brain and restore neurological function at a very distal time-point (over 1 year) after TBI in mice, challenging the long-held dogma that the treatment window for saving brain tissue from chronic degeneration and impaired cognitive functioning is limited to an acute time period immediately following injury.^42^

To properly design a laboratory model of bTBI, it is important to emphasize the unique aspects of blast-wave exposure. A blast wave is a supersonic disturbance characterized by a shock front causing a nearly instantaneous “step” increase in all gas dynamic conditions, such as static pressure, having a rise-time of about 1 nsec. As the shock front reflects and diffracts around the body within about 1 msec, complex patterns of principal and shear stress are imparted as traveling waves through tissue, yet there is usually no external evidence of wounding. Stress rates and stress enhancement at tissue interfaces or boundaries are expected to be as injurious as stress amplitude itself. It is important to consider the phenomenon of rise-time when studying primary blast injury.

Damage from primary blast injury is due to the unique loading process from the supersonic shock front as it reflects and diffracts around an object. The shock front rise-time is ∼1 nsec, and the shock front then diffracts around a human in less than 0.5 msec. This process imparts complex stress waves that enhance damage at material interfaces, most distinctly to air-backed organs such as lung, gut, and ear. There is no true comparator for this manner of physical insult. Typical car-crash impact to the head, for example, is focal in nature and has a load rise-time that is thousands of times longer than a shock front from blast. Humans, as well as many inert structures, are actually highly resilient to hydrostatic compression when loading is quasi-steady, even if rapidly time-variant, because the material is able to equilibrate throughout the process. For example, whereas a pressure change of 20 atm or 300 psi over a few minutes can be endured by a free-diver, a shockwave loading of only 2 atm will destroy the eardrum and entail substantial risk of death from lung and gut damage. Organs surrounded by fluid, such as the brain and spinal cord, may be subjected to stress concentrations as the wave passes through them. In the brain, this involves sharply transient pressure changes, possibly including partial vacuum or underpressure conditions capable of causing fluid cavitation. These stresses acutely and chronically damage the brain, with primary neuropathology of diffuse axonal injury and cytoskeletal changes.

**FIG. 8.**
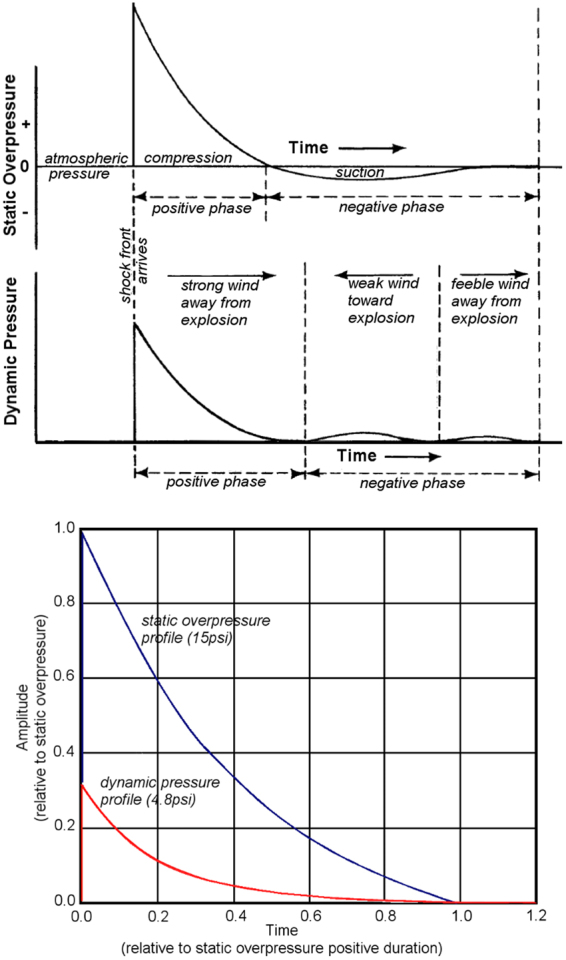
Description of blast-wave profiles for static and dynamic pressure (adapted with permission from Glasstone and Dolan^51^).

**FIG. 9.**
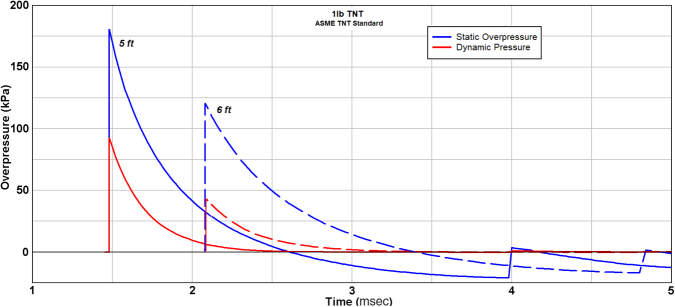
Description of blast-wave profiles for static and dynamic pressure for the case of 1 lb (0.454 kg) TNT at distances of 5 and 6 ft standoff (1.52, 1.83 m). (reprinted with permission from American National Standards Institute^50^).

With regard to effects on a specimen, a true blast wave as depicted in Figures 8 and 9 will reflect and diffract around a target, subjecting it to a transient shock front with a rise-time of ∼1 nsec as it sweeps fully around the exposed surfaces. The process of shock diffraction itself occurs in less than 1 msec for an object the scale of a human head, and from 50 to 100 μsec for a specimen the size of a rat. By the nature of shock diffraction, the loading entirely envelops all exposed surfaces of a target with particularly high reflected-shock conditions on surfaces facing the incident blast. Following the diffraction phase, the target is exposed globally to a quasi-steady decay of static pressure combined with a relatively small component of blast-wind effect. By comparison, notwithstanding a negligible precursor shock, impingement of the jet flow in this case has a rise-time on the order of 1.5 msec and subjects a target to extreme drag forces in a mode similar to high-speed aerodynamics. The flow energy is almost entirely from quasi-steady dynamic pressure.

The two conditions of incident blast primarily responsible for mechanical damage are static and dynamic pressure. Briefly, static pressure is the “crushing” action of the blast, whereas dynamic pressure is the kinetic energy of the flow, also known as the blast-wind effect. The energy partition between static and dynamic pressure changes with blast intensity. For a weak acoustic blast <10 kPa in static overpressure, the dynamic pressure is negligible. For blast with peak static overpressure of 103 kPa (15 psi), the peak dynamic pressure is about 33 kPa (4.8 psi). However, for a strong blast of 480 kPa (69 psi) approaching the edge of a typical explosive fireball, the peak amplitudes of static and dynamic pressure are about equal. Another distinction between these flow conditions is that the shape and duration of the waveforms for dynamic pressure and static overpressure differ, with dynamic pressure decaying more abruptly and lasting longer. Figure 9 is an example excerpt from a American National Standards Institute publication,^48^ showing how the two blast conditions change from a 5-ft to 6-ft standoff from a 1-lb TNT blast.

Historically, blast-wave phenomenology has been widely studied with respect to nuclear blast in the 1940s, and numerous references describe the technical specifications for the gas dynamic conditions relevant to nuclear or high-explosive blast.^46–53^ As shown in Figure 8, the distinctive feature of the traveling wave in free-field conditions (without any target obstruction) is the shock front denoting a nearly step-function increase in all the gas dynamic properties (static pressure, density, temperature, and flow velocity). Immediately following the shock front, the flow conditions decay to below ambient levels before gradually returning to ambient. The incident free-field static overpressure condition, particularly peak level and duration or impulse, is traditionally used to cite the level of blast exposure.^54^ However, it is important to note that the static overpressure is not the actual loading that an exposed object would experience. Rather, the incident wave reflects and diffracts around a target, causing widely variant transient pressure loading over its exposed surfaces.

The respective damage or injury potential of the static and dynamic pressure conditions is thus dependent on the nature of the target's shape, materials, and structural make-up. It is also strongly dependent on scale, meaning the blast wavelength compared with the “characteristic length” of the target. Objects that are small relative to the scale of the blast are strongly affected by the throwing effect of blast wind. For example, a human with a torso diameter of 0.3 m subjected to a 35-kPa blast of 5-msec duration will only feel jolted by acceleration forces, but will likely suffer serious eardrum injury due to the static pressure of the blast wave. However, a human subjected to a 35-kPa blast of 500 msec duration, which translates to about 150 m in wavelength (the scale of explosion from a tactical nuclear weapon or ammunition depot explosion), will be thrown several meters and suffer serious impact trauma, an effect due to the dynamic pressure or blast wind. This latter effect is also known as tertiary blast injury.

**FIG. 10.**
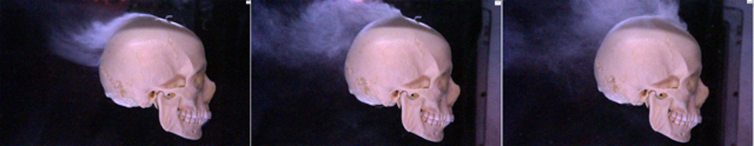
Frame sequence from high-speed imaging of blast-wave exposure of ‘free-flight’ human-skull replica showing flow patterns; negligible motion was imparted for a blast of 125 kPa and 6 ms duration. (reprinted with permission from Ritzel and colleagues^44^).

The effect of blast scaling relevant to bTBI was investigated by studies in which different-sized spheres, ranging from the size of a table-tennis ball to a soccer ball, were subjected to the same standardized blast wave of 125 kPa amplitude and 6 msec duration, representative of a roadside IED-scale event. All spheres were imparted with an initial “kick-off” velocity from the reflection and diffraction of the shock front. However, results showed that an unusual fluid-dynamic phenomena was invoked by the flow gradients developed from the blast diffraction phase. This caused the spheres to be increasingly decelerated by the blast wind with increasing size.^44^ Supplementary studies with ballasted human-skull replicas demonstrated similar effects with non-spherical shapes as well, as shown in Figure 10. The acceleration forces on a human skull are minor for this scale of blast, with displacement being only a few millimeters. Therefore, the transient crushing action of static overpressure, particularly during the initial reflection and diffraction of the shock front, is the dominant injury mechanism.

## Conclusion

A comprehensive series of experiments has been conducted with the jet-flow OPC, along with modeling by CFD, to evaluate the injurious flow conditions. The results show that the exposure conditions are those of a very high-speed jet-flow impingement rather than simulation of explosive blast. The severe impulsive loading is clearly capable of inflicting neurotrauma in a systematic manner, for example, conceptually similar to laboratory models of controlled cortical impact or fluid percussion. The jet-flow OPC apparatus is therefore useful for discovery and validation of putative TBI therapies, as well as investigation of underlying pathophysiological processes in the brain after TBI.

## Supplementary Material

Supplemental data

Supplemental data

## Abbreviations Used

2D: two-dimensional
3D: three-dimensional
AD: Alzheimer's disease
bTBI: blast-mediated traumatic brain injury
CFD: computational fluid dynamics
HSV: high-speed video
IED: improvised explosive device
MDD: major depressive disorder
mmTBI: multi-modal traumatic brain injury
OPC: overpressure chamber
RR: relative risk
TBI: traumatic brain injury

## References

[1] Masel, B.E., and DeWirr, D.S. (2010). Traumatic brain injury: a disease process, not an event. J. Neurotrauma 27, 1529–1540 20504161 10.1089/neu.2010.1358

[2] Johnson, V.E., Stewart, J.E., Begbnie, F.D., Trojanowski, J.Q., Smith, D.H., and Stewart W. (2013). Inflammation and white matter degeneration persist for years after a singel traumatic brain injury. Brain 136, 28–42 23365092 10.1093/brain/aws322 PMC3562078

[3] Johnson, V.E., Stewart, W., and Smith, D.H. (2013). Axonal pathology in traumatic brain injury. Exp. Neurol. 246, 35–43 22285252 10.1016/j.expneurol.2012.01.013 PMC3979341

[4] Vincent, A.S., Roebuck-Spencer, T.M., and Cernich, A. (2014). Cognitive changes and dementia risk after traumatic brain injury: implications for aging military personnel. Alzheimers Dement. 10 (3 Suppl.), S174–S187 10.1016/j.jalz.2014.04.006 24924669

[5] Taylor, C.A., Bell, J.M., Breiding, M.J., and Xu, L. (2017). Traumatic brain injury-related emergency department visits, hospitalizations, and deaths: United States, 2007 and 2013. MMWR Surveill. Summ. 66, 1–16 10.15585/mmwr.ss6609a1 PMC5829835 28301451

[6] Faul, M., and Coronado, C. (2015). Epidemiology of traumatic brain injury. Handb. Clin. Neurol. 127, 3–13 25702206 10.1016/B978-0-444-52892-6.00001-5

[7] Ma, V.Y., Chan, L., and Carruthers, K.J. (2014). Incidence, prevalence, costs, and impact on disability of common conditions requiring rehabilitation in the United States: stroke, spinal cord injury, traumatic brain injury, multiple sclerosis, osteoarthritis, rheumatoid arthritis, limb loss, and back pain. Arch. Phys. Med. Rehabil. 95, 986–995 24462839 10.1016/j.apmr.2013.10.032 PMC4180670

[8] Centers for Disease Control and Prevention. (2015). Report to Congress on traumatic brain injury in the United States: epidemiology and rehabilitation. National Center for Injury Prevention and Control, Division of Unintentional Injury Prevention. Atlanta, GA

[9] Jourdan, C., Azouvi, P., Genet, F., Selly, N., Josseran, L., and Schnitzler, A. (2018). Disability and health consequences of traumatic brain injury: national prevalence. Am. J. Phys. Med. Rehabil. 97, 323–331 29016402 10.1097/PHM.0000000000000848

[10] Li, Y., Li, Y., Li, X., Zhang, S., Zhao, J., Zhu, X., and Tian, G. (2017). Head injury as a risk factor for dementia and Alzheimer's disease: a systematic review and meta-analysis of 32 observational studies. PLoS One 12, e0169650 28068405 10.1371/journal.pone.0169650 PMC5221805

[11] Sullivan, P., Petitti, D., and Barbaccia, J. (1987). Head trauma and age of onset of dementia of the Alzheimer type. JAMA 257, 2289–2290 10.1001/jama.1987.03390170045014 3573227

[12] Gedye, A., Beattie, B.L., Tuokko, H., Horton, A., and Korsarek, E. (1989). Severe head injury hastens age of onset of Alzheimer's disease. J. Am. Geriatr. Soc. 37, 970–973 2794319 10.1111/j.1532-5415.1989.tb07283.x

[13] Nemetz, P.M., Leibson, C., Naessens, J.M., Beard, M., Kokmen, E., Annegers, J.F., and Kurland, L.T. (1999). Traumatic brain injury and time to onset of Alzheimer's disease: a population-based study. Am. J. Epidemiol. 149, 32–40 9883791 10.1093/oxfordjournals.aje.a009724

[14] Guo, Z., Cupples, L.A., Kurz, A., Auerbach, S.H., Volicer, L., Chui, H., Green, R.C., Sadovnick, A.D., Duara, R., DeCarli, C., Johnson, K., Go, R.C., Growden, J.H., Haines, J.L., Kukull, W.A., and Farrer, L.A. (2000). Head injury and the risk of AD in the MIRAGE study. Neurology 54, 1316–1323 10746604 10.1212/wnl.54.6.1316

[15] Plassman, B.L., Havlik, R.J., Steffens, D.C., Helms, M.J., Newman, T.N., Drosdick, D., Phillips, C., Gau, B.A., Welsh-Bohmer, K.A., Burke, J.R., Guralnik, J.M., and Breitner, J.C. (2000). Documented head injury in early adulthood and risk of Alzheimer's disease and other dementias. Neurology 55, 1158–1166 11071494 10.1212/wnl.55.8.1158

[16] Lyketsos C.G., and Olin J. (2002). Depression in Alzheimer's disease: overview and treatment. Biol. Psych. 52, 243–252 10.1016/s0006-3223(02)01348-3 12182930

[17] Rapoport, M.J. (2012). Depression following traumatic brain injury: epidemiology, risk factors and management. CNS Drugs 26, 111–121 22296315 10.2165/11599560-000000000-00000

[18] Mainio A., Kyllonen T., Viilo K., Hakko, H., Sarkioja, T., and Rasanen, P. (2007). Traumatic brain injury, psychiatric disorders, and suicide: a population-based study of suicide victims during the years 1988–2004 in Northern Finland. Brain Inj. 21, 851–855 17676442 10.1080/02699050701504265

[19] Warden, D. (2006). Military TBI during the Iraq and Afghanistan wars. J. Head Trauma Rehabil. 21, 398–402 16983225 10.1097/00001199-200609000-00004

[20] Hoge, C.W., McGurk, D., Thomas, J.L., Cox, A.L., Engel, C.C., and Castro, C.A. (2008). Mild traumatic brain injury in U.S. soldiers returning from Iraq. N. Engl. J. Med. 358, 453–463 18234750 10.1056/NEJMoa072972

[21] DePalma, R.G., and Hoffman, S.W. (2018). Combat blast related traumatic brain injury (TBI): decade of recognition: promise of progress. Behav. Brain Res. 340, 102–105 27555540 10.1016/j.bbr.2016.08.036

[22] Belmont, P.J., McCriskin, B.J., Sieg, R.N., Burks, R., and Schoenfeld, A.J. (2012). Combat wounds in Iraq and Afghanistan from 2005 to 2009. J. Trauma Acute Care Surg. 73, 3–12 22743366 10.1097/TA.0b013e318250bfb4

[23] Kapur, G.B., Hutson, H.R., Davis, M.A., and Rice, P.L. (2005). The United States twenty-year experience with bombing incidents: implications for terrorism preparedness and medical response. J. Neurotrauma 59, 1536–1544 10.1097/01.ta.0000197853.49084.3c 16394919

[24] Tate, C.M., Wang, K.K.W., Eonta, S., Zhang, Y., Carr, W., Tortella, F.C., Hayes, R.L., and Kamimori, G.H., (2013). Serum brain biomarker level, neurocognitive performance, and self-reported symptoms changes in soldiers repeatedly exposed to low-level blast: a breacher pilot study. J. Neurotrauma 30, 1620–1630 23687938 10.1089/neu.2012.2683

[25] Kamimori, G.H., LaValle, C.R., Eonta, S.E., Carr, W., Tate, C., and Wang, K.K.W. (2018). Longitudinal investigation of neurotrauma serum biomarkers, behavioral characterization, and brain imaging in soldiers following repeated low-level blast exposure (New Zealand Breacher Study). Mil. Med. 183, 28–33 29635591 10.1093/milmed/usx186

[26] Military Health System. U.S. Department of Defense. DoD worldwide numbers for traumatic brain injury. https://dvbic.dcoe.mil/dod-worldwide-numbers-tbi (Last accessed 61, 2020).

[27] DePalma, R.G., Cross, G.M., Beck, L.B., and Chandler, D. (2016). Epidemiology of mTBI (mild traumatic brain injury) due to blast: history, DOD/VA data bases: challenges and opportunities. NATO Res. Tech. Org. 2011.

[28] DePalma, R.G., Burris, D.G., Champion, H.R., and Hidgson, M.J. (2005). Blast injuries. N. Engl. J. Med. 352, 1335–1342 15800229 10.1056/NEJMra042083

[29] Okie, S. (2005). Traumatic brain injury in the war zone. N. Engl. J. Med. 352, 2043–2047 15901856 10.1056/NEJMp058102

[30] Bhattacharjee, Y. (2008). Shell shock revisited: solving the puzzle of blast trauma. Science 319, 406–408 18218877 10.1126/science.319.5862.406

[31] Vázquez-Rosa, E., Watson, M.R., Sahn, J.J., Hodges, T.R., Schroeder, R.E., Cintrón-Pérez, C.J., Shin, M.K., Yin, T.C., Emery, J.L., Martin, S.F., Liebl, D.J., and Pieper, A.A. (2019). Neuroprotective efficacy of a sigma 2 receptor/TMEM97 modulator (DKR-1677) after traumatic brain injury. ACS Chem. Neurosci. 10, 1595–1602 30421909 10.1021/acschemneuro.8b00543 PMC6862717

[32] Dutca, L.M., Stasheff, S.F., Hedberg-Buenz, A., Rudd, D.S., Batra, N., Blodi, F.R., Yorke, M.S., Yin, T., Shankar, M., Herlein, J.A., Naidoo, J., Morlock, L., Williams, N., Kardon, R.H., Anderson, M.G., Pieper, A.A., and Harper, M.M. (2014). Early detection of subclinical visual damage after blast-mediated TBI enables prevention of chronic visual deficit by treatment with P7C3-S243. Invest. Ophthalmol. Vis. Sci. 55, 8330–8341 25468886 10.1167/iovs.14-15468 PMC5102342

[33] Harper, M.M., Rudd, D., Meyer, K.J., Kanthasamy, A.G., Anantharam, V., Pieper, A.A., Vázquez-Rosa, E., Shin, M.K., Chaubey, K., Koh, Y., Evans, L.P., Bassuk, A.G., Anderson, M.G., Dutca, L., Kudva, I.T., and John, M. (2020). Identification of chronic brain protein changes and protein targets of serum autoantibodies after blast-mediated traumatic brain injury. Heliyon 6, e03374 32099918 10.1016/j.heliyon.2020.e03374 PMC7029173

[34] Yin, T.C., Voorhees, J.R., Genova, R.M., Davis, K.C., Madison, A.M., Britt, J.K., Cintrón-Pérez, C.J., McDaniel, L., Harper, M.M. and Pieper, A.A. (2016). Acute axonal degeneration drives development of cognitive, motor, and visual deficits after blast-mediated traumatic brain injury in mice. eNeuro, ENEURO.0220-16.2016 10.1523/ENEURO.0220-16.2016 PMC5086797 27822499

[35] Yin, T.C., Britt, J.K., De Jesus-Cortes, H., Lu, Y., Genova, R.M., Khan, M.Z., Voorhees, J.R., Shao, J., Katzman, A.C., Huntington, P.J., Wassink, C., McDaniel, L., Newell, E.A., Dutca, L.M., Naidoo, J., Cui, H., Bassuk, A.G., Harper, M.M., McKnight, S.L., Ready, J.M., and Pieper, A.A. (2014). P7C3 neuroprotective chemicals block axonal degeneration and preserve function after traumatic brain injury. Cell Rep. 8, 1731–1740 25220467 10.1016/j.celrep.2014.08.030 PMC4206693

[36] Courtney, A., and Courtney, M. (2015). The complexity of biomechanics causing primary blast-induced traumatic brain injury: a review of potential mechanisms. Front. Neurol. 6, 221 26539158 10.3389/fneur.2015.00221 PMC4609847

[37] Jean, A., Nyein, M.K., Zheng, J.Q., Moore, D.F., Joannopoulos, J.D., and Radovitzky, R. (2014). An animal-to-human scaling law for blast-induced traumatic brain injury risk assessment. Proc. Natl. Acad. Sci. USA 111, 15310–15315 25267617 10.1073/pnas.1415743111 PMC4217421

[38] Rafaels, K.A., Bass, C.R.D., Panzer, M.B., Salzar, R.S., Woods, W.A., Feldman, S.H., Walilko, T., Kent, R.W., Capehart, B.P., Foster, J.B., Derkunt, B., and Toman, A. (2012). Brain injury risk from primary blast. J. Trauma Acute Care Surg. 73, 895–901 22836001 10.1097/TA.0b013e31825a760e

[39] Bass, C.R., Panzer, M.B., Rafaels, K.A., Wood, G., Shridharani, J., and Capehart, B. (2011). Brain injuries from blast. Ann. Biomed. Eng. 40, 185–202 22012085 10.1007/s10439-011-0424-0

[40] Beamer, M., Tummala, S.R., Gullotti, D., Kopil, C., Gorka, S., Abel, T., Bass, C.R.D., Morrison 3rd, B., Cohen, A.S., and Meaney, D.F. (2016). Primary blast injury causes cognitive impairments and hippocampal circuit alterations. Exp. Neurol. 283 (Pt. A), 16–28 10.1016/j.expneurol.2016.05.025 PMC5062598 27246999

[41] Kabu, S., Jaffer, H., Petro, M., Dudzinski, D., Stewart, D., Courtney, A., Courtney, M., and Labhasetwar, V. (2015). Blast-associated shock waves result in increased brain vascular leakage and elevated ROS levels in a rat model of traumatic brain injury. PLoS ONE 10, e0127971 26024446 10.1371/journal.pone.0127971 PMC4449023

[42] Vázquez-Rosa, E., Shin, M.-K., Dhar, M., Chaubey, K., Cintrón-Pérez, C., Tang, X., Liao, X., Miller, E., Koh, Y., Schroeder, R., Emery, J., Yin, T.C., Harper, M.M., Jain, M.K., and Pieper, A.A. (2020). P7C3-A20 treatment one year after traumatic brain injury in mice repairs the blood-brain barrier, arrests chronic neurodegeneration, and restores cognition. Proc. Natl. Acad. Sci. USA 117, 27667–27675 33087571 10.1073/pnas.2010430117 PMC7959512

[43] Martec Limited, Chinook Manual, Part 1: Chinook Overview, SM-07-01 Rev. 3, Feb 2008, Martec Limited, 1888 Brunswick St. Suite 400, Halifax, NS, Canada B3J 3J8

[44] Ritzel, D.V., Van Albert, S., Sajja, V., and Long, J. (2018). Acceleration from short-duration blast. Shock Waves 28, 101–114

[45] Needham, C.E., Ritzel, D.V., Rule, G.T., Wiri, S., and Young, L. (2015). Blast testing issues and TBI: experimental models that lead to wrong conclusions. Front. Neurol 6, 72 25904891 10.3389/fneur.2015.00072 PMC4389725

[46] Taylor, G.I. (1950). The formation of a blast wave by a very intense explosion. I. Theoretical discussion. Proc Royal Soc London. Series A, Mathematical and Physical Sciences 201, 59–174

[47] Bethe, H.A., Fuchs, K., Hirshfelder, J.O., Magee, J.L., Peierls, R.E., and von Neumann, J. (1947). Blast Wave. Los Alamos Scientific Laboratory, LA-2000, Physics &Mathematics (TID-4500, 13th Ed, Suppl).

[48] Sedov, L.I. (1946). Propagation of strong shock waves. J Appl Math Mech 10, 241–250

[49] Brode, H.L., (1959). Blast Wave from a Spherical Charge. Phys Fl 2, No.2

[50] American National Standards Institute. (2011). Estimating Air Blast Characteristics for Single Point Explosions in Air, with a Guide to Evaluation of Atmospheric Propagation and Effects, ANSI/ASA S2.20-1983

[51] Dewey McMillin & Associates, Airblast: software describing the physical properties of blast waves from explosions in air; http://www.blastanalysis.com/abframe.htm (last accessed 6/1/2020)

[52] Hyde, D.W. (1988). Microcomputer Programs CONWEP and FUNPRO, Applications of TM 5-855-1, ‘Fundamentals of Protective Design for Conventional Weapons’ (User's Guide). https://apps.dtic.mil/dtic/tr/fulltext/u2/a195867.pdf. (last accessed 6/1/2020)

[53] Glasstone, S. and Dolan, P.J., (Eds.) (1977). The Effects of Nuclear Weapons (3rd Ed., 1977), US Dept of Defense & Energy Research & Development Agency. https://www.dtra.mil/Portals/61/Documents/NTPR/4-Rad_Exp_Rpts/36_The_Effects_of_Nuclear_Weapons.pdf (last accessed 6/1/2020)

[54] Bowen, I.G, Fletcher, E.R., and Richmond, D.R. (1968). Estimate of man's tolerance to the direct effects of air blast. DASA2113, Defense Atomic Support Agency

